# A Quick and Simple Way of Making Dies and Swaging Plates from Plaster Impressions

**Published:** 1904-09

**Authors:** Harry W. Bates

**Affiliations:** Assistant Demonstrator Dental Department University of Denver.


### A QUICK AND SIMPLE WAY OF MAKING DIES AND SWAGING PLATES FROM PLASTER IMPRESSIONS.

By HARRY W. BATES, D. D. S., Assistant Demonstrator Dental Department University of Denver.

(Written for *The American Journal of Dental Science.*)

(This method was demonstrated as a clinic at the June meeting of the Colorado State Dental Association, 1903.)

The plaster impression having been taken as usual, paying particular attention to having the plaster run high up under lip and cheeks, you may proceed as follows: Build up with soft moldine (which has been previously softened by rolling with bottle in glycerine) or plaster whenever the impression is low so as to keep metal from running over and to give thickness to die for strength. Place on Bunsen burner and allow to dry for one hour or till fairly well dried out. Melt metal having enough for two dies. Pour into plaster impression slowly patting metal into deep portions and undercuts with wad of cotton covered with old rubber dam. When sufficiently cool, separate, which will be very easy as metal contracts slightly upon cooling. Repeat the operation so as to have two metal dies. After which varnish impression and run up plaster cast for final swaging. Work over the dies to start as over any metal cast. When the sides are forced down and surplus trimmed off, cover plate and die with rubber dam and place in Parker swager using finest mustard seed shot made. After hitting a few blows, remove from swager and after going through pickling bath with plate anneal and repeat in swager till perfect in fit, changing to other die as the first die becomes bruised and dinged. Giving it a final swaging on the plaster cast made last. When trimmed and fitted to mouth the teeth may be put on as the individual operator may desire. The metal I use is made out of 3 parts of lead, 5 parts tin, 3 parts of bismuth and 1 part of cadmium. This I find works very satisfactorily and is the lowest price low fusing metal made. In melting metal, first melt lead, and fuse tin, then cadmium and bismuth into them as the bismuth oxidizes very easily and wastes. It will be necessary to replace the bismuth so lost by adding to original mass small pieces occasionally. Always dissolve lead off surface of plate before annealing by passing through some acid pickle. One composed of sulphuric acid and water, one part of each, is very good.

---

### VULCANIZABLE GUTTA-PERCHA IN DENTAL PROSTHESIS.

By J. DUNKLIN REYNOLDS, D. D. S., Marietta, Ga.

(Written for *The American Journal of Dental Science.*)

